# From continental strategy to country implementation: a document-based scorecard for assessing health security and sovereignty readiness in Africa

**DOI:** 10.1080/16549716.2026.2735716

**Published:** 2026-09-28

**Authors:** Augustus Osborne, Camilla Bangura, Abdirasak Sharif Ali, Umaru Sesay, Morie Vandi

**Affiliations:** a Institute for Development, Western Area, Freetown, Sierra Leone; b Department of Biological Sciences, School of Basic Sciences, Njala University, Freetown, Sierra Leone; c Department of Microbiology and Laboratory Sciences, Faculty of Medicine and Health Sciences, SIMAD University, Mogadishu, Somalia; d Africa Centres for Disease Control and Prevention (Africa CDC), Addis Ababa, Ethiopia; e Ministry of Health, Western Area, Freetown, Sierra Leone

**Keywords:** Africa Centres for Disease Control and Prevention, policy document analysis, national public health institutes, health system building blocks, domestic health financing

## Abstract

**Background:**

The Africa Health Security and Sovereignty (AHSS) Agenda of the Africa Centres for Disease Control and Prevention (Africa CDC) provides a continental framework for strengthening health security and sovereignty. As implementation begins, no document-based readiness assessment instrument exists to monitor progress against AHSS pillars and goals, limiting efforts to identify bottlenecks and target assistance.

**Objective(s):**

To develop and apply a document-based scorecard for assessing policy readiness for AHSS implementation across selected African countries.

**Methods:**

We conducted a qualitative policy analysis using systematic document review, deductive framework development, structured coding, and scorecard synthesis. The corpus comprised 48 documents: the AHSS Agenda, the African Union Roadmap to 2030, WHO AFRO guidance, and national policies from 12 named African countries in 5 regional economic community zones. A 10-dimension scorecard based on AHSS pillars and WHO building blocks was applied on a 3-level scale. A second author recoded the corpus independently; materials are openly deposited.

**Results:**

Readiness was most consistently reflected in governance and strategic planning and least in financing specificity, digital health architecture, and laboratory and pharmaceutical systems. AHSS-relevant provisions appeared in 3 of 12 financing strategies, 3 of 10 pharmaceutical policies, and 2 of 12 plans, indicating strategic intent but weak operational readiness across the 10 dimensions.

**Conclusions:**

The AHSS/WHO document-based scorecard is a feasible and replicable instrument for monitoring readiness. It identifies governance and implementation gaps and helps Africa CDC, WHO AFRO, governments, and partners target technical assistance. Periodic application would strengthen implementation monitoring, cross-setting comparison, and accountability.

## Paper Context

- **Main findings:** A document-based readiness scorecard applied to 48 policy documents from 12 African countries found stronger readiness in governance and strategic planning, but persistent gaps in financing specificity, digital health architecture, laboratory systems, and pharmaceutical systems.
- **Added knowledge:** This study provides a feasible and replicable tool that translates the Africa Health Security and Sovereignty Agenda pillars and World Health Organization health-system building blocks into a structured approach for assessing policy readiness across countries.
- **Global health impact for policy and action:** Periodic use of the scorecard can help the Africa Centres for Disease Control and Prevention, the World Health Organization Regional Office for Africa, governments, and development partners identify implementation bottlenecks, target technical assistance, and strengthen accountability for progress.

The Africa Health Security and Sovereignty (AHSS) Agenda of the Africa Centres for Disease Control and Prevention (Africa CDC) advances continental health security governance in Africa [1]. Drawing on lessons from the COVID-19 pandemic and the 2014 to 2016 West Africa Ebola outbreak, it has five pillars: reshaping global health architecture; institutionalising continental health security preparedness, prevention, and response; predictable domestic, innovative, and blended financing; digital transformation; and local manufacturing [1,2]. It advances Africa’s New Public Health Order by promoting expanded local manufacturing, stronger public health institutions, and a self-reliant, sovereign health security architecture [3]. As the most operationally specific continental health security framework produced by an African intergovernmental institution, its implementation affects all 55 African Union (AU) member states [3,4]. Following the African Union Assembly’s endorsement of the transition to the AHSS Agenda in February 2026, a critical gap became apparent: no validated, document-based readiness assessment instrument exists for the systematic, replicable monitoring of country-level implementation against the AHSS pillars and goals [5]. Africa CDC, WHO AFRO, national governments, and development partners need such a tool to track progress, align support, identify policy gaps, and target financing and technical assistance toward missing readiness dimensions in national strategies [6].

This methodological gap is not incidental. The AHSS Agenda is a complex, multidimensional framework that spans health security, sovereignty, and system strengthening across conventional assessment boundaries [1]. Existing instruments, including the Joint External Evaluation under the International Health Regulations framework and the Global Health Security Index, were designed for different purposes and normative frameworks [7–9]. The Joint External Evaluation does not address sovereignty, financing, digital health, or pharmaceutical manufacturing, which are central to the AHSS Agenda [6,8]. The Global Health Security Index does not map AHSS pillars or WHO building blocks in the integrated manner that AHSS implementation monitoring requires [9,10]. Therefore, a purpose-built readiness instrument is needed.

The case for a document-based approach is both methodologically and practically grounded. National policy documents, including health sector strategic plans, financing and digital health strategies, and laboratory and pharmaceutical policies, are the main instruments through which governments express their health system governance commitments and implementation intentions [11,12]. Assessing their alignment with the AHSS Agenda is necessary for monitoring implementation and identifying governance gaps in technical assistance [6,13]. It is replicable, transparent, applicable across contexts, and scalable without primary data collection [14].

The WHO health system building blocks framework provides a structure for the scorecard [15]. Its six blocks, namely service delivery, health workforce, information systems, medical products and technologies, financing, and leadership and governance, map onto the AHSS Agenda’s dimensions, enabling the assessment of AHSS pillar alignment and health system coherence. Integrating both frameworks yields an assessment that covers both agendas and grounds the scorecard in a tradition familiar to planners and technical assistance providers [15,16] (Figure 1).

**Figure 1.**
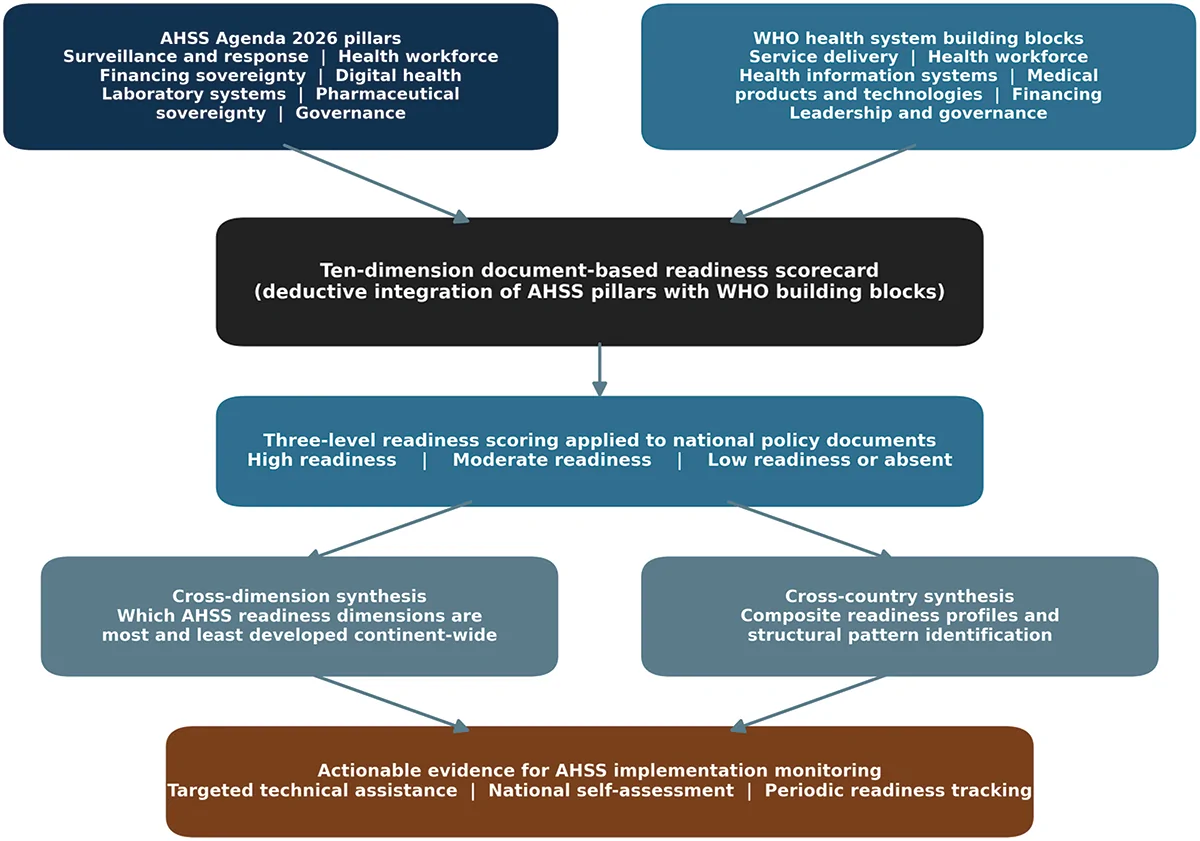
Conceptual framework for the document-based AHSS readiness scorecard. The 10-dimension scorecard was derived deductively by integrating Africa CDC AHSS Agenda pillars with WHO health system building blocks, applied to national policy documents using a 3-level readiness scale, and synthesised across dimensions and countries to produce actionable evidence for implementation monitoring. AHSS, Africa health security and sovereignty Agenda; WHO, World Health Organization.

Prior literature documents readiness assessment tools in global health security, notably the Joint External Evaluation, which assesses capacities across 19 technical areas, and the Global Health Security Index, which uses document-based and open-source scoring across 195 countries [8,10]. In Uganda, qualitative health policy research examined stakeholder participation in district priority setting [17], while comparative policy analysis and framework synthesis assessed national health strategy coherence and implementation specificity in African contexts [6,18].

Closer comparators than these sit within the Africa CDC and the International Association of National Public Health Institutes assessment tradition, and any new instrument should be positioned against them. Desta and colleagues assessed 12 fully established national public health institutes across corporate governance, health information systems, workforce development, emergency response coordination, and public health research and used the results to identify candidate regional centres of excellence [19]. The Staged Development Tool of the International Association of National Public Health Institutes offers a maturity-staged self-assessment built around 20 discussion guides that cover leadership, financing, workforce, laboratory, surveillance, and research and has been used by more than 35 institutes to plan capacity development [20]. The Africa CDC event-based surveillance scorecard, distributed to all 55 African Union member states and completed by 49 of them between July 2022 and April 2023, measures a single capacity domain in depth rather than the breadth of a national policy framework [21]. Africa CDC is also developing continental health information exchange policies and standards, with a maturity assessment piloted in test member states, work that bears directly on the digital architecture and interoperability dimension assessed here [22]. Table 1 sets out how each of the 10 dimensions relates to the domains that these instruments already cover.

**Table 1.** Relationship between the 10 scorecard dimensions and the domains covered by existing Africa CDC, International Association of national public health institutes, and global health security assessment instruments.

Scorecard dimension	Coverage by Africa CDC and International Association of National Public Health Institutes instruments	Coverage by the Joint External Evaluation and the Global Health Security Index	Position of the present dimension
AHSS pillar alignment and strategic coherence	Not covered. The Staged Development Tool assesses strategic direction within each discussion guide, but against institute maturity rather than against a continental agenda [20]	Not covered. Both instruments predate the AHSS Agenda and score capacity against the International Health Regulations and an open-source index framework [7,9]	New. Scores whether national policy instruments map to the AHSS pillars and carry provisions for implementing them
Governance and institutional arrangements	Covered. Corporate governance is a scored domain in the national public health institute assessment [19], and leadership and governance run through the internal-facing discussion guides [20]	Partly covered. The Joint External Evaluation assesses national legislation, policy, and financing for International Health Regulations capacities [7]	Retained rather than new. Assessed here from policy text rather than institute self-report, and specifically for coordination with line ministries, subnational authorities, and regional bodies
Financing specificity and resource mobilisation	Not a named domain. Both instruments record dependence on external funding as a constraint rather than scoring budget architecture [19,20]	Not covered in the sovereignty sense. The Global Health Security Index scores financing indicators, but not domestic substitution for donor financing [9,10]	New. Scores AHSS-relevant budget lines, domestic revenue mobilisation instruments, and donor substitution strategies in national financing strategies and medium-term expenditure frameworks
Health workforce planning and deployment	Covered. Public health workforce development is a scored domain [19] and a discussion guide topic [20]	Covered. Human resources is a Joint External Evaluation technical area [7]	Retained rather than new. Assessed for health security surge and deployment provisions written into national workforce and strategic plans
Digital health architecture and interoperability	Partly covered and actively developing. Health information management systems is a scored domain [19], and Africa CDC is piloting a health information exchange maturity assessment in test member states [22]	Partly covered. Surveillance and reporting technical areas assess data systems, but not interoperability standards or cross-border data governance [7,9]	Retained and narrowed. Scores whether national digital health strategies link architecture to AHSS surveillance, emergency response, and cross-border data sharing. Overlaps directly with the Africa CDC health information exchange work and should be aligned with it as those standards are issued
Laboratory and diagnostic system strengthening	Covered. Laboratory is a discussion guide topic [20] and features in the institute capacity assessment [19]	Covered. National laboratory system is a Joint External Evaluation technical area [7]	Retained rather than new. Assessed for biosafety, surge capacity, and financing provisions in national laboratory policy, including linkage to the medium-term expenditure framework
Pharmaceutical and supply chain governance	Not a named domain in either instrument [19,20]	Not covered. Neither instrument assesses domestic manufacturing or pharmaceutical sovereignty [7,9]	New. Scores domestic manufacturing strategy, supply chain resilience, stockpiling, and the financing attached to them
Health security and surveillance integration	Covered in depth. The Africa CDC event-based surveillance scorecard assesses this capacity across member states and is the closest comparator for this dimension [21]; emergency response coordination is a scored domain in the institute assessment [19]	Covered. Surveillance, reporting, and emergency response are Joint External Evaluation technical areas [7]	Retained rather than new. Assessed from policy commitments rather than self-reported capacity, and read against the AHSS requirements. Results should be read alongside the event-based surveillance scorecard rather than in place of it
Implementation pathway clarity	Not covered as a scored construct. The Staged Development Tool supports planning through its workshop process but does not score the delivery architecture written into national policy [20]	Not covered by either instrument [7,9]	New in this form. Scores whether a commitment carries a sequence of activities, a responsible institution, attached resources, and phasing. Concerns how delivery is specified, not how it is verified
Accountability and monitoring arrangements	Partly covered. Quality and impact are cross-cutting domains within each discussion guide [20]	Partly covered. Both instruments publish country scores, which is itself an accountability function, but neither scores national monitoring architecture [7,9]	Retained and narrowed. Scores whether indicators, targets, reporting obligations, and review points exist and are tied to AHSS-relevant performance rather than to general health sector performance. Concerns how delivery is verified, not how it is specified

AHSS, Africa Health Security and Sovereignty Agenda; Africa CDC, Africa Centres for Disease Control and Prevention.

Read against these instruments, the contribution of the present scorecard is narrower than a general claim of novelty, and correspondingly easier to defend. Six of the 10 dimensions cover ground that the Africa CDC, Staged Development Tool, Joint External Evaluation, and Global Health Security Index instruments already address in some form, although none of them scores that ground against the AHSS pillars. What is new here is the method and three of its dimensions. The method is document-based: it reads what governments have committed to in the current policy instruments, whereas the comparator tools record self-reported or directly observed institutional capacity. The two forms of evidence answer different questions, and a country can score well on one and poorly on the other. The three dimensions are: AHSS pillar alignment, which no existing instrument assesses because no existing instrument predates the Agenda; financing specificity and resource mobilisation; and pharmaceutical and supply chain governance. The latter two follow the sovereignty pillars introduced by the AHSS Agenda and are not named domains in any of the comparator instruments. The remaining dimensions are retained because a readiness profile that omitted workforce, laboratory, surveillance, or accountability would not be usable for planning and not because they are original to this instrument.

This paper reports the development and piloting of a document-based scorecard for AHSS implementation readiness. The scorecard was developed from the AHSS Agenda and WHO building blocks framework, refined through preliminary document reading, and piloted across a purposive sample of national policy documents from African countries. This study assessed policy document content, readiness scoring methodology, and policy coherence, but not health system performance, expenditure outturns, or service delivery outcomes. Its objectives were to develop a 10-dimension scorecard, pilot it across African countries, identify dimensions most and least reflected in policy documents, and provide a practical tracking instrument.

## Methods

### Study design

This study employed a qualitative comparative policy analysis design, combining a systematic document review with deductive framework development, structured coding, and comparative scorecard synthesis [23]. This design was selected because it enables rigorous and replicable construction and piloting of a readiness assessment instrument from publicly available policy documents without requiring field access, expert interviews, or primary data collection [11,13]. Framework development and deductive coding are well-established methods for constructing structured analytical tools from normative frameworks and systematically applying them across heterogeneous document corpora [14,24]. Comparative scorecard synthesis enables the aggregation of coding results into a structured readiness profile that is comparable across country contexts and analytically actionable for implementation monitoring [6,14]. Reporting followed the Standards for Reporting Qualitative Research [25].

### Document corpus

The corpus was assembled through purposive selection to ensure the coverage of all relevant governance levels and document types. The inclusion criteria required that documents be current health governance instruments produced by recognised governmental or intergovernmental bodies with direct relevance to AHSS implementation dimensions, published or revised between 2022 and 2026. Documents were identified through the official repositories of the Africa CDC, AU Commission, WHO AFRO, national ministries of health, and relevant line ministries, supplemented by targeted searches of institutional websites and grey literature databases. The identification, screening, and inclusion processes are summarised in Figure 2.

**Figure 2.**
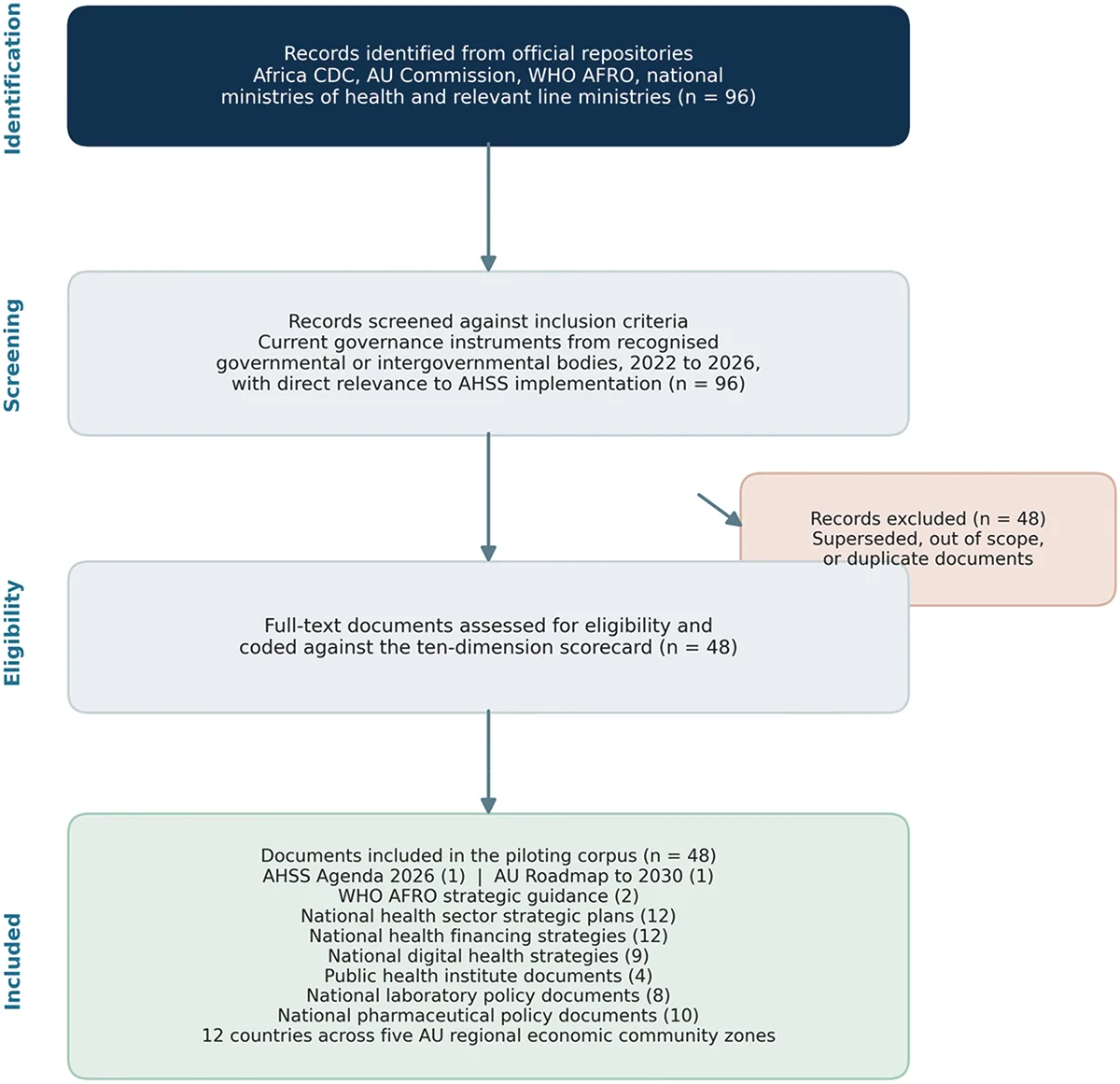
Identification, screening, and inclusion of policy documents in the pilot corpus. AHSS, Africa health security and sovereignty Agenda; AU, African union; WHO AFRO, World Health Organization regional office for Africa.

The corpus comprises nine document categories. The first was the Africa CDC AHSS Agenda, the primary reference for scorecard development [1]. The second is the African Union’s Roadmap to 2030 and Beyond, which provides a broader context on health system strengthening, health security, sustainable financing, and local manufacturing [26]. The third category comprised WHO AFRO guidance, including the Africa Health Workforce Investment Charter and the framework for resilient health systems, universal health coverage, and health security [27,28]. The fourth and fifth sections comprise national health sector strategic plans and financing strategies from sampled African countries across the AU regional economic community zones. The sixth category comprised national digital health strategies where they were publicly available. The seventh to ninth categories comprised public health institute, laboratory, and pharmaceutical policy documents.

The final corpus comprised 48 documents in 9 categories. National documents were obtained from 12 countries in 5 AU regional economic community zones, selected for variation in income level, health system maturity, donor dependency, AHSS-relevant governance reforms, and stages of public health and health security institution building. The identities of the 12 countries and the full list of the 48 assessed documents with the issuing institution, the year of publication or revision, and the source location for each are given in the deposited study materials so that the corpus can be reconstructed and the coding checked independently.

### Scorecard development

The scorecard was developed in three stages. First, a 10-dimensional framework was built from the AHSS Agenda pillars and WHO building blocks, mapping each AHSS pillar to the WHO dimensions and identifying indicators for assessing readiness in policy documents [1,16]. The dimensions were as follows: (1) AHSS pillar alignment and strategic coherence in national plans; (2) governance and institutional arrangements for coordination and accountability; (3) financing specificity and resource mobilisation, including budgets, domestic revenue mobilisation, and donor dependency management; (4) health workforce planning and deployment, including surge capacity and security provisions; (5) digital health architecture and interoperability, including systems, standards, and data governance; (6) laboratory and diagnostic system strengthening; (7) pharmaceutical and supply chain governance; (8) health security and surveillance integration; (9) implementation pathway clarity; and (10) accountability and monitoring arrangements. Dimensions 9 and 10 are related but distinct, and the scoring criteria keep them apart. Implementation pathway clarity asks whether a policy document sets out how a commitment will be delivered, that is, whether it names the sequence of activities, the institution responsible for each, the resources attached, and the phasing over time. Accountability and monitoring arrangements ask whether the document sets out how delivery will be observed and answered for, that is, whether it specifies indicators, targets, reporting obligations, and review points, and whether these are tied to AHSS-relevant performance rather than to general health sector performance. A plan must therefore be clear about delivery and silent about verification, and several documents in the corpus were exactly that.

In the second stage, the provisional framework was refined by reading 10 corpus documents, with coding categories adjusted to reflect the encountered content [29]. Refinements added sub-indicators for digital governance and pharmaceutical sovereignty and clarified the scoring criteria for distinguishing strategic intent from operational readiness.

In the third stage, the scoring criteria for each dimension were finalised using a three-level readiness scale. High readiness requires specific operational provisions with implementation architecture, including timelines, responsible institutions, and budget provisions. Moderate readiness acknowledged the dimension with specificity but without full operationalisation. Low readiness or absence indicated only general references without substantive governance content or complete absence. Table 2 presents this framework.

**Table 2.** The 10-dimension AHSS readiness scorecard framework: scoring criteria, document indicators, and illustrative piloting examples.

Scorecard dimension	AHSS pillar or WHO building block	Scoring criteria	Document indicators	Illustrative piloting examples
AHSS pillar alignment and strategic coherence	AHSS all pillars; WHO leadership and governance	High: explicit AHSS pillar mapping with implementation provisions. Moderate: AHSS with partial alignment. Low or absent: no AHSS reference or generic health security language only	National health sector strategic plan; Africa CDC partnership framework references	Eight of 12 national plans reference AHSS pillars; four specify implementation alignment with AHSS goals
Governance and institutional arrangements	AHSS governance pillar; WHO leadership and governance	High: institutional structures, coordination mechanisms, and accountability frameworks are specified. Moderate: Structures specified, coordination, or accountability absent. Low or absent: generic governance language without institutional specificity	National health sector strategic plan; public health institute policy documents	Seven national plans specify health security governance structures; four specify coordination mechanisms
Financing specificity and resource mobilisation	AHSS financing sovereignty; WHO financing building block	High: AHSS-relevant budget lines, domestic revenue mobilisation, and donor substitution strategies specified. Moderate: Health security financing referenced with partial specificity. Low or absent: generic financing language without AHSS-relevant provisions	National health financing strategy; medium-term expenditure framework	Three of 12 financing strategies specify AHSS-relevant budget provisions; two specify domestic substitution strategies
Health workforce planning and deployment	AHSS health workforce pillar; WHO workforce building block	High: AHSS-relevant cadre deployment, surge capacity, and health security workforce provisions specified. Moderate: Health security workforce referenced with partial specificity. Low or absent: generic workforce language without health security provisions	National health sector strategic plan; national health workforce strategy	Five national plans specify health security workforce provisions; three specify surge capacity arrangements
Digital health architecture and interoperability	AHSS digital health pillar; WHO health information building block	High: integrated health information system, interoperability standards, and AHSS surveillance linkage were specified. Moderate: Digital health architecture specified without AHSS linkage. Low or absent: no digital health strategy or generic digital health language	National digital health strategy; health information system policy	Three of nine digital health strategies link architecture to AHSS health security functions; four specify interoperability standards
Laboratory and diagnostic system strengthening	AHSS laboratory pillar; WHO medical products building block	High: Laboratory network, biosafety, quality assurance, surge capacity, and financing provisions specified. Moderate: Laboratory network specified without biosafety, surge capacity, or financing provisions. Low or absent: no laboratory policy or generic laboratory language	National laboratory policy document; biosafety framework	Four of eight laboratory documents specify biosafety provisions; two specify surge capacity; one links to medium-term expenditure framework
Pharmaceutical and supply chain governance	AHSS pharmaceutical sovereignty pillar; WHO medical products building block	High: domestic manufacturing strategy, supply chain resilience, stockpiling, and financing provisions. Moderate: supply chain strengthening referenced without manufacturing or financing specificity. Low or absent: generic essential medicine language without sovereignty provisions	National pharmaceutical policy; supply chain strategic plan	Three of ten pharmaceutical documents reference domestic manufacturing; one specifies a concrete investment strategy
Health security and surveillance integration	AHSS health security pillar; WHO service delivery and information building blocks	High: integrated disease surveillance, event-based surveillance, and emergency response provisions specified and linked to the AHSS requirements. Moderate: Surveillance provisions specified without AHSS linkage or emergency response integration. Low or absent: generic surveillance language	National health sector strategic plan; public health institute strategic plan	Six national plans specify integrated surveillance provisions; three link surveillance to AHSS emergency response requirements
Implementation pathway clarity	AHSS implementation framework; WHO all building blocks	High: Implementation timelines, responsible institutions, resource requirements, and phasing logic were specified. Moderate: timelines specified without resources or institutional provisions. Low or absent: no implementation roadmap or generic activity lists This dimension assesses how a commitment will be delivered, not how delivery will be verified.	National health sector strategic plan; AHSS implementation framework references	Four of 12 national plans specify credible implementation roadmaps; four specify indicative timelines only
Accountability and monitoring arrangements	AHSS accountability framework; WHO leadership and governance	High: monitoring and evaluation framework with AHSS-specific indicators, targets and reporting obligations specified. Moderate: monitoring framework present without AHSS-specific indicators. Low or absent: no monitoring framework or generic monitoring language This dimension assesses how delivery will be observed, reported, and answered for, independently of whether a delivery pathway is specified.	National health sector strategic plan; monitoring and evaluation framework	Two national plans specify AHSS-linked monitoring indicators; five reference health security within broader monitoring frameworks

AHSS, Africa Health Security and Sovereignty Agenda; WHO, World Health Organization.

### Piloting procedures

The final scorecard was applied to all the 48 documents in the corpus. Each document was coded against the 10 dimensions using the scoring criteria [24]. Where more than one document type was available for a country, the dimension scores were aggregated to produce a composite readiness profile. Piloting assessed the applicability of the scorecard across document types and the readiness patterns present in the sample.

### Coding reliability

Coding was carried out by the lead author and verified by a second author, who recoded the full corpus against the same written criteria without sight of the first coder’s scores. The two sets of scores were compared dimension by dimension, and every disagreement was resolved by returning to the source document and recording the reason for the final score in a coding log. That log, the written criteria, and the completed country by dimension coding matrix are deposited with the other study materials, so the judgement behind each score can be inspected and, if a reader wishes, recomputed.

Chance-corrected agreement statistics such as Cohen’s kappa were not computed prospectively for this pilot, and we report that as a limitation rather than present a coefficient calculated after the event. Two considerations shaped the original design. The first is that the unit on which readiness is reported is a country by dimension judgement, giving 120 units on a three-level ordinal scale with markedly uneven marginal distributions; a coefficient estimated on so few units under those conditions is unstable and can be low even where observed agreement is high [30]. The second is that this study set out to establish whether the instrument could be applied at all, and at that stage, the more informative reliability question was which criteria produced disagreement and why, which the coding log records directly. Neither consideration is a substitute for the reported coefficient. Any subsequent application of the scorecard should therefore double code a defined subsample prospectively, report percentage agreement and a chance-corrected coefficient for each dimension, and use the pattern of disagreement to sharpen the criteria that generate it. The deposited materials make that possible without reference to the authors.

### Analytical procedures

A comparative analysis was conducted at two levels. Cross-dimension findings identified which AHSS and WHO building block dimensions were most and least reflected in the national policy documents. Cross-country composite readiness profiles were compared to assess variations in readiness and alignment with AHSS goals. The analysis identified structural patterns, recurring gaps, and high-readiness cases, but not country-specific rankings. The findings assessed only the document content and readiness scoring methodology.

### Reflexivity and rigour

The research team included public health, health policy, and biomedical researchers from Sierra Leone, where health security institutions are being built. This positionality shaped our stance, which is attentive to governance realities in resource-constrained settings. To limit bias, the criteria were documented in advance, coding used explicit indicators, and the corpus was independently recoded by a second author, with disagreements resolved by returning to the source documents and recording the reasons for the final score [31,32]. The materials needed to replicate the study are openly deposited rather than held by the authors.

### Ethical considerations

This study involved the analysis of publicly available policy documents and did not require an ethical review or informed consent procedures.

### Patient and public involvement

Patients and members of the public were not involved in the design, conduct, reporting, or dissemination planning of this study because it was a document-based policy analysis of publicly available government and institutional policy documents and did not involve human participants or primary data collection.

## Results

### Overview of scorecard development and piloting

A 10-dimension scorecard framework was developed and piloted across the 48-document corpus, showing its applicability across contexts. All 10 dimensions were assessable from national policy documents, although the evidence varied across dimensions and countries. Continental and regional documents, the AHSS Agenda, and the WHO AFRO strategic guidance provided normative content and standards for assessing national readiness. National documents varied in readiness, with governance and strategic planning being better represented than financing, digital health, and laboratory and pharmaceutical systems (Figure 3). Across the corpus, strategic intent coincided with weak operational readiness, with implications for AHSS monitoring and assistance targeting (Figure 4).

**Figure 3.**
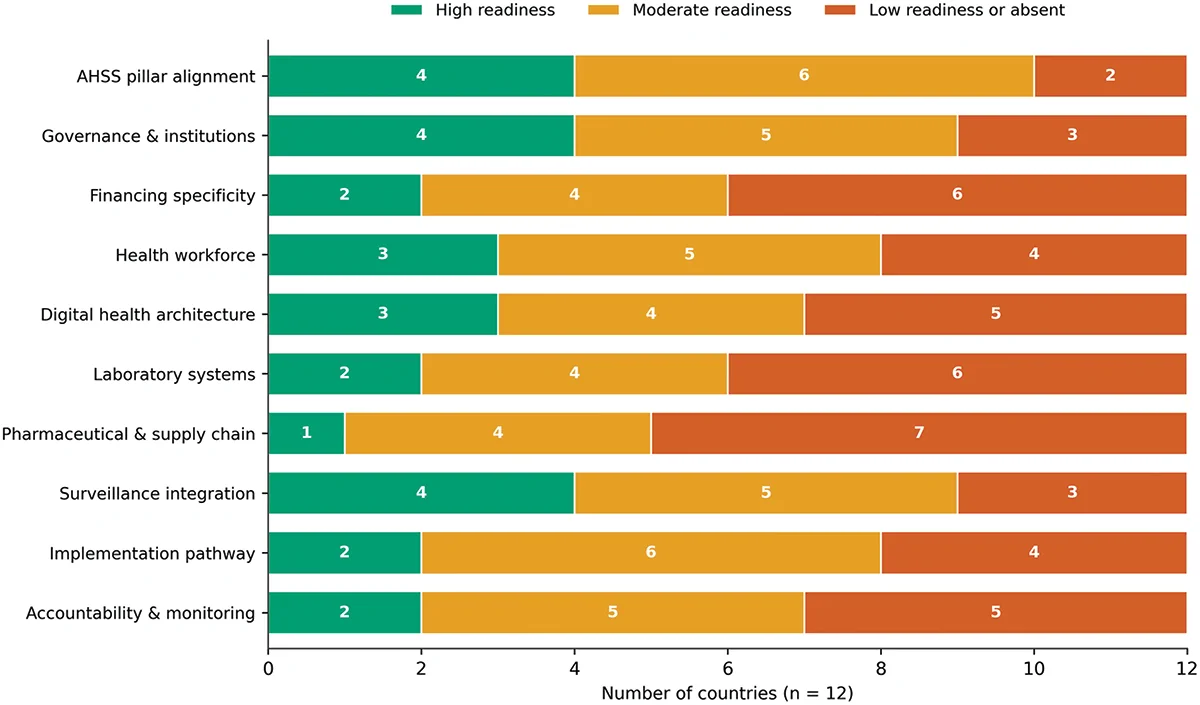
Distribution of country readiness levels (high, moderate, and low or absent) across the 10 scorecard dimensions in the 12-country purposive sample (*n* = 12 countries).

**Figure 4.**
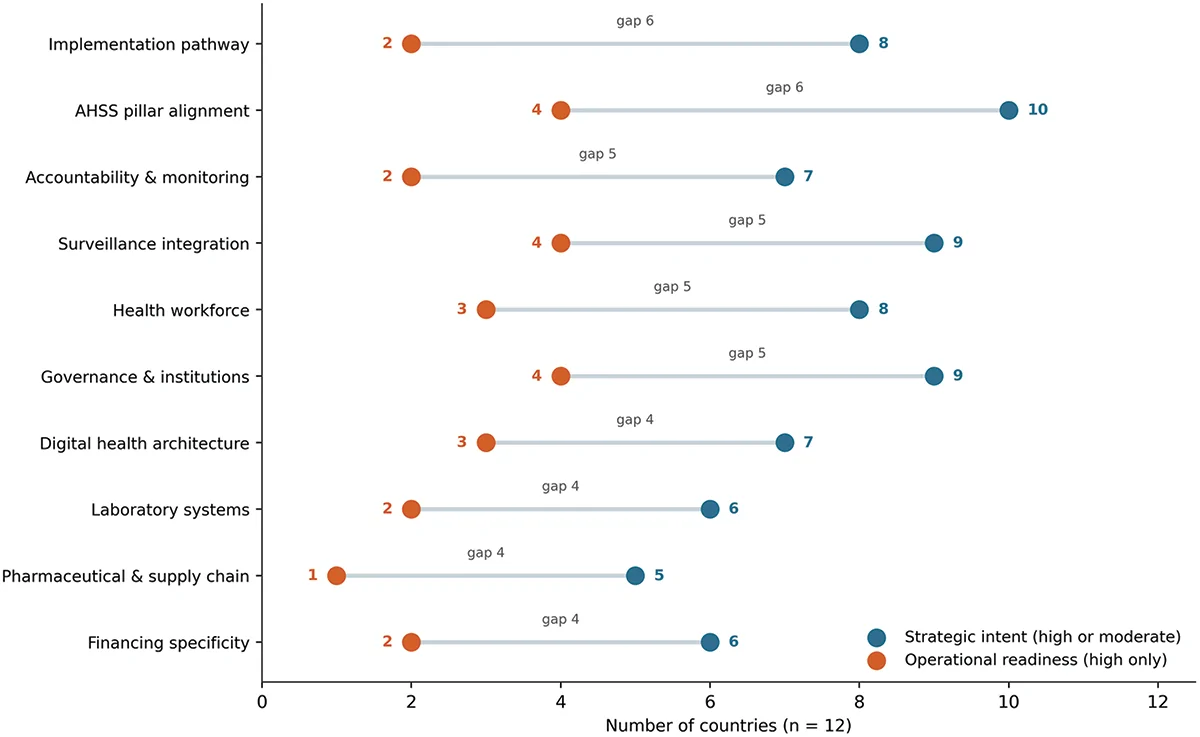
The readiness gap between strategic intent (countries with high or moderate readiness) and operational readiness (countries with high readiness) for each scorecard dimension, ordered by gap size.

### Governance and strategic planning readiness is the most consistently developed dimension, but institutional coordination provisions are frequently absent

Governance and strategic planning showed readiness across national documents, consistent with the African health governance literature, which shows that strategic planning capacity exceeds implementation capacity [6,11]. Of the 12 national health strategic plans, 10 prioritised health security, 8 referenced AHSS pillars or Africa CDC partnership frameworks, and 7 specified governance arrangements. This reflects investment after the 2014 to 2016 West Africa Ebola outbreak and the COVID-19 pandemic response [33,34]. However, the provisions for institutional coordination varied. Most plans identified governance structures, but only four specified how these would coordinate with line ministries, subnational authorities, and regional bodies for AHSS implementation. Accountability frameworks, including performance indicators, reporting obligations, and review mechanisms, were present in six plans, but only three linked them to domestic monitoring systems; the rest referenced donor-funded frameworks, creating a limitation for sustained AHSS implementation [34,35].

The AHSS Agenda sets continental priorities for health security governance, preparedness, response, financing, digital transformation, and local manufacturing [1]. The African Union’s Roadmap to 2030 and beyond complements these efforts [36]. The challenge is the gap between continental provisions and national institutional coordination capacities.

### Financing specificity and resource mobilisation provisions are consistently weak and AHSS-relevant budget architecture is largely absent

Financing specificity was a low-readiness dimension across the national document corpus, with AHSS-relevant budget provisions absent from most of the reviewed national health financing strategies. Of the 12 strategies, all 12 prioritised health security, but only 3 specified budget provisions for AHSS-relevant functions. Despite the longstanding Abuja commitment to allocate 15% of government expenditure to health [37], the remaining nine endorsed health security financing in principle without specifying any budget instruments.

Domestic revenue mobilisation provisions for AHSS-relevant functions appeared in all five national financing strategies, but their specificity varied. Two documents specified mechanisms and timelines, including earmarked health security levies and national insurance provisions with explicit health security benefits for older adults. Three others referenced mobilisation without any fiscal mechanisms. Donor dependency management provisions appeared in four documents, but only two specified domestic substitution strategies, leaving most countries without governance responses to contracting health security financing [38,39].

The lack of AHSS-relevant budget architecture in national financing documents is the most consequential financing readiness gap. Public health institute operations, laboratory network maintenance, surveillance infrastructure, and health security workforce deployment are recurrent functions that require predictable domestic budget provisions for sustainable operations [40,41]. Strategy endorsements cannot replace national budget line provisions or medium-term expenditure framework allocations. This reflects a broader structural gap in African health financing governance [42].

## Digital health architecture and interoperability provisions are present but fragmented and rarely linked to health security functions

Digital health readiness showed moderate and uneven development across the corpus. National digital health strategies were available for 9 of the 12 countries, reflecting expanded post-pandemic policy development across Africa [43,44]. Of these nine strategies, seven specified integrated health information system architectures with interoperability provisions, and six referenced digital health platforms and data exchange frameworks. This progress exceeded the pre-pandemic baseline and reflected regional efforts and the Africa CDC’s digital transformation agenda [29,45].

However, three of the nine national digital health strategies linked digital health architecture to AHSS health security functions. The other six framed digital health around service delivery and health management information systems without specifying support for AHSS surveillance, event-based detection, emergency response coordination, or cross-border data sharing. Data governance frameworks relevant to AHSS interoperability, including cross-border sharing, real-time surveillance integration, and emergency health information management, appeared in all four strategies but were specified in only two strategies. This fragmentation creates a gap in AHSS implementation. Although the AHSS Agenda includes digital transformation as one of its five pillars [1], strategies that do not link service delivery systems with surveillance, laboratory information, and cross-border reporting may provide partial support [46,47].

## Laboratory and pharmaceutical system provisions are the least operationally developed AHSS readiness dimensions

Laboratory and pharmaceutical system provisions were the least developed readiness dimensions in the national corpus, with gaps between continental commitments and national operational provisions in Africa. National laboratory policy documents were available for 8 of the 12 cases and pharmaceutical policy documents for 10; however, specificity varied, and alignment with the AHSS laboratory and pharmaceutical sovereignty provisions was weak. Of the eight laboratory policy documents reviewed, six specified network development plans with tiered referral structures and quality assurance provisions in place. However, biosafety and biosecurity provisions aligned with AHSS requirements appeared in only four documents, surge capacity for diagnostic response in two, and laboratory financing provisions in three, with only one linked to the medium-term expenditure framework. This gap threatens laboratory system sustainability because donor-dependent networks remain vulnerable to external financing contractions, affecting health programme areas [48,49].

Pharmaceutical sovereignty provisions aligned with the AHSS goals were the weakest dimensions in the corpus. All 10 national pharmaceutical policy documents referenced essential medicine access and supply chain strengthening in some form. However, domestic pharmaceutical manufacturing provisions, a central AHSS sovereignty goal advanced by the Partnerships for African Vaccine Manufacturing [50], were present in only three documents, and only one specified a manufacturing investment strategy with financing and timelines. Supply chain resilience provisions were present in four documents but were operationally specified in two. This wide alignment gap reflects the investment and governance reforms required for domestic pharmaceutical manufacturing capacity [51,52].

## Implementation pathway clarity and accountability arrangements are inconsistent, and monitoring frameworks are rarely linked to AHSS indicators

Implementation pathway clarity and accountability showed low to moderate readiness nationally, varying by country-specific differences. Of the 12 national health sector strategic plans, 8 specified timelines for health security commitments, but only 4 linked them to resources and responsible institutions in credible roadmaps. The other four presented timelines as indicative, without financing, institutional responsibility, or monitoring provisions needed for credibility [53,54].

Accountability and monitoring arrangements for AHSS-relevant commitments were present in all seven national documents but were linked to AHSS-specific indicators in only two documents. The remaining five documents referenced health security monitoring within broader health sector monitoring and evaluation frameworks that did not disaggregate AHSS-relevant performance indicators from general health system performance. This lack of AHSS-specific monitoring architecture means that implementation progress against AHSS goals cannot be systematically tracked by national monitoring systems in most countries, creating a significant gap in the evidence base for AHSS implementation accountability [55,56].

The two countries with the most advanced implementation pathways and accountability provisions shared a common characteristic: both had participated in Africa CDC-supported health security planning processes that included explicit AHSS indicator development and monitoring framework design. This is an observation on 2 cases within a purposive sample of 12. It cannot establish that the engagement produced the readiness rather than that the countries already investing in health security governance were the ones that sought the engagement, and the design of this study cannot separate the two. We record it as a hypothesis for testing in a larger and differently constructed sample, and we do not treat it as a finding elsewhere in this paper [1,57].

The distribution of readiness across the full country sample by dimension is summarised in Table 3, and the gap between strategic intent and operational readiness across all 10 dimensions is shown in Figure 4. Across the corpus, the number of countries reaching high readiness ranged from one (pharmaceutical and supply chain governance) to four (AHSS pillar alignment, governance and institutional arrangements, and health security and surveillance integration), while the number reaching only low readiness or absent status reached seven for pharmaceutical and supply chain governance and six for both financing specificity and laboratory systems.

**Table 3.** Piloting results across the 12-country purposive sample, by scorecard dimension.

Scorecard dimension	High readiness (countries)	Moderate readiness (countries)	Low readiness or absent (countries)	Key documentary evidence pattern	Notable gaps
AHSS pillar alignment and strategic coherence	4	6	2	Explicit AHSS pillar mapping in recently revised national plans; generic health security language in older strategies	Two countries with pre-2023 strategies show no AHSS alignment; recently revised plans show stronger uptake
Governance and institutional arrangements	4	5	3	Public health institute mandates and health security committee provisions present in majority; coordination mechanisms absent in most	Intersectoral coordination mechanisms absent in eight of 12 countries; accountability frameworks linked to domestic systems in three only
Financing specificity and resource mobilisation	2	4	6	AHSS-relevant budget provisions in two countries with national health insurance frameworks; generic financing language dominant	AHSS-relevant budget lines absent in nine of 12 countries; donor substitution strategies in two countries only
Health workforce planning and deployment	3	5	4	Health security workforce provisions in countries with Africa CDC partnership programmes; surge capacity absent in majority	Surge capacity provisions in three countries; health security workforce financing provisions in two; community health worker health security integration in three
Digital health architecture and interoperability	3	4	5	Integrated health information system architectures in countries with recent digital health strategy revisions; AHSS surveillance linkage rare	AHSS surveillance linkage absent in six of nine digital health strategies; cross-border data sharing provisions in two countries only
Laboratory and diagnostic system strengthening	2	4	6	Tiered laboratory network plans in majority of countries with laboratory policies; biosafety and surge capacity provisions rare	Biosafety provisions in four countries; surge capacity in two; laboratory financing linked to medium-term expenditure framework in one country only
Pharmaceutical and supply chain governance	1	4	7	Domestic manufacturing strategy in one country with active pharmaceutical investment programme; supply chain strengthening referenced broadly	Domestic manufacturing provisions in three countries; concrete investment strategies in one; supply chain resilience financing in two
Health security and surveillance integration	4	5	3	Integrated surveillance provisions in countries with established public health institutes; emergency response integration rare	Emergency response integration with AHSS requirements in three countries; event-based surveillance provisions in four; cross-border surveillance coordination in two
Implementation pathway clarity	2	6	4	Credible implementation roadmaps in two countries with Africa CDC-supported planning processes; indicative timelines dominant	Resource-linked implementation timelines in four countries; responsible institution designation in six; phasing logic in three
Accountability and monitoring arrangements	2	5	5	AHSS-linked monitoring indicators in two countries with Africa CDC partnership frameworks; broader health security monitoring in five	AHSS-specific indicators in two countries; domestic monitoring system linkage in three; donor-funded monitoring frameworks dominant in seven

Country groups reflect predominant patterns across purposive samples rather than individual country assessments. Country names are given in the methods. AHSS, Africa Health Security and Sovereignty Agenda.

## Discussion

### Principal findings and their significance for AHSS implementation monitoring

This study developed and piloted the first instrument that scores national policy documents against the Africa CDC AHSS Agenda, showing that a 10-dimensional framework based on AHSS pillars and WHO health system building blocks can be applied consistently across policy document corpora to generate comparative readiness profiles. The results revealed that readiness was strongest in governance and strategic planning and weakest in financing specificity, digital health architecture, and laboratory and pharmaceutical systems. Across dimensions, strong strategic intent is paired with weak operational readiness, shaping the priorities for technical assistance and implementation support [6,11,58].

The finding that only 2 country cases demonstrated high readiness across more than 5 of the 10 scorecard dimensions is not an indictment of African health governance capacity; it reflects the gap between the normative ambition of the AHSS Agenda and the governance architecture that most national health systems have yet to develop. The scorecard identifies the most consequential readiness gaps and governance investments likely to yield the greatest gains in AHSS implementation capacity, making them more analytically actionable than narrative assessments of policy alignment [34,57].

### Interpretation of the readiness gap between strategic intent and operational specificity

The gap between strategic intent and operational specificity revealed by the scorecard across all 10 dimensions is understood as a governance translation problem, rather than a political commitment deficit. African governments have demonstrated their commitment to health security and sovereignty by endorsing the AHSS Agenda, participating in Africa CDC partnership programmes, and revising national health sector strategic plans to reflect continental priorities [1,3]. The challenge is not commitment but the governance instruments needed to convert it into operational readiness: budget provisions, coordination mechanisms, implementation roadmaps, accountability frameworks, and technical capacity for AHSS implementation. This problem has a dimension common to all readiness dimensions. Continental frameworks specify operational requirements, and national plans endorse them in principle; however, the governance instruments needed to convert these endorsements into operational capacity remain absent or incomplete. There is a need for dimension-specific technical assistance to develop the missing instruments [56,59,60].

Whether sustained external engagement is what converts commitment into governance instruments is a question this study can raise but cannot answer. The implementation science literature identifies sustained engagement, technical assistance, and accountability mechanisms as drivers of that conversion [58,59], and the two country cases described in the results are consistent with it. Two cases drawn purposively, however, cannot distinguish that explanation from the simpler one that countries already further along were the ones that sought the engagement. The question is worth testing directly, in a sample large enough to compare countries with and without such engagement while holding constant the governance investments that preceded it.

### Implications for AHSS implementation monitoring

The scorecard developed in this study has direct practical value as a monitoring instrument for tracking AHSS implementation. Its 10 dimensions map onto the AHSS Agenda’s implementation requirements, its scoring criteria are transparent and replicable, and its document-based design enables periodic application without requiring field access or primary data collection. Applied annually or biennially to updated national policy document corpora, the scorecard would generate a time-series evidence base on AHSS readiness trends that are currently absent from the continental implementation monitoring architecture [1,58].

Several refinements could strengthen the monitoring utility of the scorecard. First, the scoring criteria should be operationalised through dimension-specific indicators that specify the documentary evidence for each readiness level more precisely than the current framework. This would reduce interpretive judgement in coding and improve inter-rater reliability. Second, the scorecard should be validated by independent research teams using the same document corpus, with discrepancies used to refine the criteria. Third, it should include a supplementary module assessing subnational policy coherence, recognising that AHSS implementation depends on subnational governance capacity [60,61]. The deposited instrument, coding manual, and coding matrix make that independent validation possible without reference to the authors, and any such replication should report percentage agreement and a chance-corrected coefficient for each dimension.

The Africa CDC is well-positioned to institutionalise the scorecard as a component of its AHSS implementation monitoring architecture. The agenda’s accountability framework calls for systematic monitoring of member state implementation progress, and the scorecard provides a structured, document-based instrument through which monitoring can be conducted at scale [1]. Integrating the scorecard into Africa CDC’s annual country assessment processes, alongside the Joint External Evaluation and other existing assessment instruments, would produce a fuller and more AHSS-specific monitoring evidence base than any single instrument alone [6,8].

### Implications for national governments, Africa CDC, WHO AFRO, and AU institutions

The pilot findings have specific and actionable implications for the principal actors in AHSS implementation governance. For national ministries of health, the scorecard identifies the governance dimensions in which readiness investments would produce the greatest gains in AHSS implementation capacity. Financing specificity, laboratory system sustainability, pharmaceutical sovereignty, and digital health and health security integration are the dimensions most consistently absent from national documents and addressing them requires deliberate governance investments that go beyond strategy document revision to encompass budget architecture reform, institutional capacity development, and intersectoral coordination [42,43,54].

For ministries of finance, the financing specificity findings highlight the importance of engaging in AHSS implementation as a fiscal planning priority. The absence of AHSS-relevant budget provisions from national health financing strategies and medium-term expenditure frameworks means that health security governance commitments lack the fiscal architecture necessary to sustain them through budget reallocation cycles [43]. Finance ministries that proactively engage with health security financing as a medium-term fiscal planning issue, rather than responding reactively to annual health budget requests, are better positioned to support the domestic financing transition that AHSS sovereignty goals require.

For the Africa CDC, the practical value of these findings lies less in the two-country observation than in what the scorecard shows about where readiness is missing across the sample. Financing specificity, pharmaceutical governance, and laboratory sustainability were weak in most of the countries assessed, and each is a dimension on which continental technical assistance can act. Whether extending the direct country engagement model to countries with low readiness would raise readiness is a proposition worth testing rather than an inference that the present evidence supports. Africa CDC is well placed to test it, by applying the scorecard before and after engagement in a defined set of member states and reporting the results [1,60].

For the WHO AFRO, workforce, resilient health systems, digital health architecture, and health security integration findings highlight complementary technical assistance opportunities. WHO AFRO’s Africa Health Workforce Investment Charter and Resilient Health Systems Framework provide regional guidance on workforce investment, primary health care, and health security [28,29]. Together with the Africa CDC Digital Transformation Strategy [48], these frameworks can inform country-level assistance by linking workforce capacity, resilient systems, and digital architecture to AHSS surveillance and response. For the AU Commission, the findings reinforce the African Union’s Roadmap to 2030 and Beyond, including health-system and governance commitments [36], and support stronger AU coordination across health, finance, development, and other sectors to address structural governance gaps [62].

### Comparison with relevant literature

The findings are consistent with and extend the literature on readiness assessment and implementation science in African contexts. The Joint External Evaluation literature found governance and planning capacities to be more developed than technical implementation capacities across African health security systems, a pattern the AHSS scorecard confirms and extends to sovereignty and financing dimensions the Joint External Evaluation does not address [6,8,9]. The Global Health Security Index literature identifies financing and laboratory provisions as weak in low-income countries, consistent with the scorecard findings [10,11]. Implementation science literature contextualises the intent versus readiness gap: normative framework development alone is insufficient without implementation support, including technical assistance and accountability mechanisms [62]. Structured scoring frameworks have shown that evidence can be generated from documents and administrative data [63–65]. The AHSS scorecard extends this approach to health security and sovereignty, demonstrating that scoring is feasible and informative for assessing readiness.

The Africa CDC and Staged Development Tool instruments discussed in the background provide the closest points of comparison for the substantive results, and reading them alongside the pilot is instructive. Desta and colleagues found that the fully established national public health institutes they assessed were held back most by inadequate infrastructure and by dependence on external funding [19], which is the institutional counterpart of what appears here as absent budget architecture in national financing strategies. The event-based surveillance scorecard found minimal or average capacity in 34 of the member states that responded [21], while the present corpus shows surveillance provisions widely present in policy but tied to AHSS emergency response requirements in only three countries. Policy commitment and reported capacity are therefore weak in the same places, which is some reassurance that a document-based reading is not simply measuring drafting quality. The continental health information exchange work reported by Tamirat and colleagues [22] is developing the standards whose absence from national digital health strategies this study records, and the two readings together suggest that the gap is one of the national adoption rather than of continental specification.

### Strengths and limitations

This study has some strengths. The scorecard was developed systematically and transparently within the AHSS Agenda and WHO building blocks, ensuring that it was AHSS-specific and coherent with the health system assessment literature. Piloting across 12 countries and 48 documents showed applicability across contexts and to documents. Its document-based design enables replicable and scalable use without field access or data collection, making it practical for periodic continental monitoring.

This study has some limitations. It assesses policy content and readiness using governance logic, not performance or implementation outcomes; the findings describe government commitments on paper rather than achievements in practice. The purposive sample of 12 countries is not representative of all 55 AU member states; therefore, the findings are illustrative patterns, not definitive national assessments. Because the corpus was restricted to available documents, relevant provisions in unpublished annexes, operational plans, or inaccessible language documents may have been missed, understating readiness. The scoring framework involves interpretive judgements that cannot be fully standardised. The corpus was independently recoded by a second author and disagreements were resolved against the source documents, but chance-corrected agreement was not computed prospectively, so the reliability of the instrument remains to be quantified in a study designed for that purpose. The observation that the two most ready countries had both engaged with Africa CDC-supported planning rests on two cases and is offered as a hypothesis rather than as evidence of effect. The scorecard does not capture the subnational governance dimensions critical for AHSS implementation in federal or decentralised systems. The political economy of implementation, including health worker organisations, private sector actors, and parliamentary oversight, is not addressed and warrants future research.

## Conclusions

The AHSS Agenda is an operationally specific continental framework for health security and sovereignty in Africa; however, its implementation requires monitoring instruments to assess country-level readiness from documents. The scorecard developed and piloted in this study provides a structured, document-based instrument for this purpose, showing applicability across contexts and generating comparative evidence on the readiness dimensions that are most and least developed in policy documents. The finding that strategic intent is stronger than operational readiness across all 10 dimensions identifies governance translation as an implementation challenge and highlights financing specificity, digital health and health security integration, laboratory sustainability, and pharmaceutical sovereignty as priorities. Scaling the scorecard, refining inter-rater reliability, and extending it to subnational governance would strengthen accountability and provide a tool for tracking agenda goals in the future.

## Supplementary Material

SRQR_checklist_AHSS_scorecard.docx

## Data Availability

All documents analysed in this study are publicly available from the official repositories of the issuing institutions, and each of the 48 assessed documents is cited in full in the reference list, where the issuing institution, the year, and the source are given. The 12 sampled countries are named in the text. No datasets were generated, and no primary data were collected from human participants. The scorecard instrument, the coding manual, the completed country by dimension coding matrix and the coding log recording disagreements and their resolution are held by the author and are available from the corresponding author on reasonable request. No restrictions apply to the reuse of these materials.

## Preprints

This manuscript has not been posted to a preprint server.
