# Supplementary material for: From continental strategy to country implementation: a document-based scorecard for assessing health security and sovereignty readiness in Africa
Source: Glob Health Action. 2026 Sep 28;19(1):2735716. doi: 10.1080/16549716.2026.2735716 (PMC13629793; doi:10.1080/16549716.2026.2735716)
Supplement: Supplementary file 1 [file ZGHA_A_2735716_SM5207.docx]

**Standards for Reporting Qualitative Research (SRQR): completed checklist**

Manuscript: From continental strategy to country implementation: a document-based scorecard for assessing health security and sovereignty readiness in Africa

Corresponding author: Abdirasak Sharif Ali. First author and guarantor: Augustus Osborne.

*Item descriptions are abbreviated from O’Brien BC, Harris IB, Beckman TJ, Reed DA, Cook DA. Standards for reporting qualitative research: a synthesis of recommendations. Acad Med. 2014;89(9):1245-51. doi:10.1097/ACM.0000000000000388.*

| **SRQR item** | **What the item requires (abbreviated)** | **Where it is reported in the manuscript** |
| --- | --- | --- |
| **Title and abstract** |  |  |
| **1. Title** | Concise description of the nature and topic of the study, identifying it as qualitative or naming the approach or data collection method. | Title page. The title names the study object (a document-based scorecard) and the qualitative design is identified in the Abstract, Methods line. |
| **2. Abstract** | Summary of key elements using the journal’s abstract format; typically background, purpose, methods, results, conclusions. | Abstract, structured under Background, Objectives, Methods, Results, Conclusions. |
| **Introduction** |  |  |
| **3. Problem formulation** | Description and significance of the problem studied; relevant theory and empirical work; problem statement. | Background, paragraphs 1 to 4. Absence of an AHSS-specific readiness instrument; limits of the Joint External Evaluation and Global Health Security Index; rationale for a document-based approach; WHO building blocks as the organising structure; prior readiness assessment literature. |
| **4. Purpose or research question** | Purpose of the study and specific objectives or questions. | Background, final paragraph. Four objectives are stated: develop a ten-dimension scorecard, pilot it across African countries, identify the dimensions most and least reflected in policy documents, and provide a tracking instrument. Also Abstract, Objectives. |
| **Methods** |  |  |
| **5. Qualitative approach and research paradigm** | The qualitative approach and guiding theory; the research paradigm; rationale. | Methods, Study design. Qualitative comparative policy analysis combining systematic document review, deductive framework development, structured coding and comparative scorecard synthesis, with rationale for choosing it over field-based designs. |
| **6. Researcher characteristics and reflexivity** | Researcher attributes, qualifications and assumptions that may influence the work, and their interaction with the questions, methods and findings. | Methods, Reflexivity and rigour. Team composition, positionality as researchers working where health security institutions are being built, and the measures taken to limit its effect on coding. |
| **7. Context** | Setting and salient contextual factors; rationale. | Background, paragraphs 1 and 2, and Methods, Document corpus. Context is the AHSS Agenda following African Union Assembly endorsement in February 2026, applied across 12 countries in five regional economic community zones. |
| **8. Sampling strategy** | How and why documents were selected; criteria for stopping sampling; rationale. | Methods, Document corpus. Purposive selection; inclusion criteria of currency, issuing body, AHSS relevance and publication or revision between 2022 and 2026; sources searched; nine document categories; 12 countries chosen for variation in income level, health system maturity, donor dependency, governance reform and institution-building stage. Screening and inclusion shown in Fig. 2. |
| **9. Ethical issues pertaining to human subjects** | Ethics approval and consent, or explanation for their absence; confidentiality and data security. | Methods, Ethical considerations, and Ethics and consent. The study analysed publicly available policy documents; no ethical review or consent was required and no human participants or human data were involved. |
| **10. Data collection methods** | Types of data collected and the collection procedures, including dates, iteration, triangulation and any changes made during the study; rationale. | Methods, Document corpus and Scorecard development. Documents retrieved from official repositories of Africa CDC, the AU Commission, WHO AFRO and national ministries, supplemented by targeted institutional and grey literature searches. The framework was revised after reading ten documents, so the iterative element is reported. |
| **11. Data collection instruments and technologies** | Instruments and devices used, and whether they changed during the study. | Methods, Scorecard development, and Table 1. The scorecard is the instrument: ten dimensions, three-level readiness scale, scoring criteria and document indicators set out in full. Changes made after the ten-document reading are described, including added sub-indicators for digital governance and pharmaceutical sovereignty. |
| **12. Units of study** | Number and relevant characteristics of the documents or events studied. | Methods, Document corpus, and Results, Overview. Final corpus of 48 documents in nine categories from 12 countries across five AU regional economic community zones, with counts by category reported throughout the Results (12 strategic plans, 12 financing strategies, nine digital health strategies, eight laboratory policies, ten pharmaceutical policies). |
| **13. Data processing** | Processing before and during analysis, including data management, coding and verification of integrity. | Methods, Piloting procedures. Each document coded against the ten dimensions using the scoring criteria; scores aggregated into a composite readiness profile where several document types were available for one country; lead-researcher coding verified by second-pass review with discrepancies resolved by re-reading. |
| **14. Data analysis** | How inferences and themes were identified and developed, and by whom; the approach used; rationale. | Methods, Analytical procedures. Two-level comparative analysis: cross-dimension identification of the dimensions most and least reflected, and cross-country composite readiness profiles. Structural patterns, recurring gaps and high-readiness cases were identified; country-specific rankings were not produced. Contributions of individual authors are given under Author contributions. |
| **15. Techniques to enhance trustworthiness** | Techniques used to strengthen credibility, such as audit trail, triangulation or independent verification; rationale. | Methods, Reflexivity and rigour, and Piloting procedures. Criteria documented in advance, coding against explicit indicators, second-pass verification with documented resolution of discrepancies, and materials available for replication. |
| **Results and findings** |  |  |
| **16. Synthesis and interpretation** | Main findings, including themes, models or integration with prior work. | Results, all five subheadings, with Table 2, Fig. 3 and Fig. 4. Governance and strategic planning are the most consistently developed dimensions; financing specificity, digital health architecture, laboratory systems and pharmaceutical sovereignty the least; strategic intent exceeds operational readiness across all ten dimensions. |
| **17. Links to empirical data** | Evidence such as quotations or text excerpts substantiating the analytic findings. | Results, throughout, and Table 1, Illustrative piloting examples column. Findings are currently substantiated by document counts and paraphrased provisions rather than by quoted text. |
| **Discussion** |  |  |
| **18. Integration with prior work, implications, transferability and contribution** | Summary of main findings; how they relate to earlier scholarship; scope of application; unique contribution. | Discussion, Principal findings; Interpretation of the readiness gap; Implications for AHSS implementation monitoring; Implications for national governments, Africa CDC, WHO AFRO and AU institutions; Comparison with relevant literature. Contribution stated as the first document-based readiness scorecard for the AHSS Agenda. |
| **19. Limitations** | Trustworthiness and limitations of the findings. | Discussion, Strengths and limitations. Policy content rather than performance; purposive sample of 12 of 55 member states; restriction to publicly available documents; interpretive judgement in scoring with inter-rater reliability not quantified; no subnational coverage; political economy not addressed. |
| **Other** |  |  |
| **20. Conflicts of interest** | Potential sources of influence on the conduct and conclusions of the study, and how they were managed. | Disclosure. The authors declare no competing interests. |
| **21. Funding** | Sources of funding and other support, and the role of funders in data collection, interpretation and reporting. | Funding info. The study received no external funding. |

**Reference**

O’Brien BC, Harris IB, Beckman TJ, Reed DA, Cook DA. Standards for reporting qualitative research: a synthesis of recommendations. Academic Medicine. 2014;89(9):1245-51. doi:10.1097/ACM.0000000000000388
